# Case Report: Stereoelectroencephalography and Stereoelectroencephalography-Guided Radiofrequency Thermocoagulation in Familial Lateral Temporal Lobe Epilepsy

**DOI:** 10.3389/fneur.2022.864070

**Published:** 2022-04-04

**Authors:** Ziqi Wei, Xiaolai Ye, Changquan Wang, Jiwen Xu, Puming Zhang, Qiangqiang Liu, Jun Zhao

**Affiliations:** ^1^School of Biomedical Engineering, Shanghai Jiao Tong University, Shanghai, China; ^2^Department of Neurosurgery, Clinical Neuroscience Center Comprehensive Epilepsy Unit, Ruijin Hospital, Shanghai Jiao Tong University School of Medicine, Shanghai, China; ^3^Clinical Neuroscience Center, Ruijin Hospital Luwan Branch, Shanghai Jiao Tong University School of Medicine, Shanghai, China

**Keywords:** superior temporal gyrus, stereoelectroencephalography-guided radiofrequency thermocoagulation, stereoelectroencephalography, familial lateral temporal lobe epilepsy, case report

## Abstract

Familial lateral temporal lobe epilepsy (FLTLE) is genetic focal epilepsy usually characterised by auditory symptoms. Most FLTLE cases can be controlled by anti-seizure medications, and to our best knowledge, there are no previous reports about stereoelectroencephalography (SEEG) used for patients with FLTLE. In this report, we present two patients with FLTLE in one family and their SEEG performances, together with ^18^F-fluorodeoxyglucose (^18^F-FDG) PET and MRI results. In case 1, fast activities originated from the right superior temporal gyrus and spread rapidly to the right anterior insular lobe and hippocampus. In case 2, there were two seizure patterns: (1) The fast activities or sharp slow waves were identified at the left superior temporal gyrus, then, sharp waves and spike waves spread in the left superior temporal gyrus; (2) There were fast activities and slow-wave oscillation originated in the left superior temporal gyrus, then, the fast activities spread in the left superior temporal gyrus and finally spread to the other sites. An SEEG-guided radiofrequency thermocoagulation was performed for both patients and one of them underwent resection surgery. Seizures are well-controlled and the patients are very satisfied with the therapeutic effects.

## Introduction

Familial lateral temporal lobe epilepsy (FLTLE) is genetic focal epilepsy usually characterised by auditory symptoms. Despite the complexity of FLTLE, it is generally considered as a benign epilepsy syndrome and routinely used anti-seizure medications can usually control seizures (1).

Stereoelectroencephalography (SEEG) is a methodology for presurgical invasive evaluation and SEEG-guided radiofrequency thermocoagulation (RFTC) can be used as an alternative to resection surgery. Using stereotactically implanted electrodes, SEEG provides neuronal electrical activities recordings in the deep brain (2) and helps locate epileptogenic zone for thermocoagulation or resection surgery (3). Most FLTLE seizures can be controlled by anti-seizure medications, and there are no previous reports about SEEG being used in the treatment of patients with FLTLE. Here we report two refractory FLTLE cases in one family. SEEG and SEEG-guided RFTC were performed for both patients and one of them underwent resection surgery. Seizures are well-controlled and the patients are very satisfied with the therapeutic effects.

## Case Report

### Case 1

A 47-year-old woman, with her grandmother and her son, was diagnosed with FLTLE. Seizures firstly occurred when she was 22, with the symptoms of a sudden loss of consciousness, daze, delayed movements, chewing, and hand groping-like movements. Genetic tests were refused.

In scalp electroencephalogram (EEG), interictal spikes and intermittent slow waves were observed in the bilateral anterior temporal region and sphenoidal electrode. Seizures were characterised by initial attenuation of background activity followed by low-voltage fast activity in the right temporal-occipital lobe, then, quickly spread to the right and left temporal lobe.

**Figure 1:**
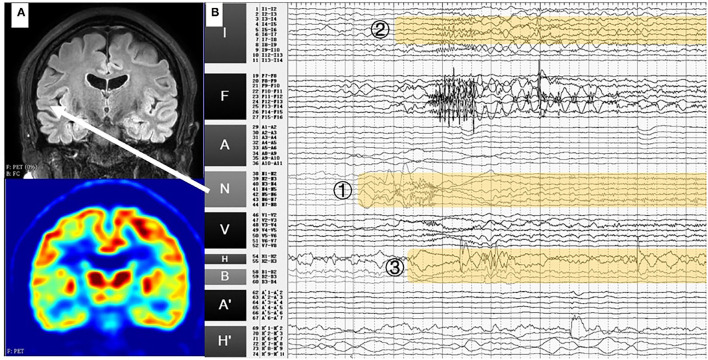
**(A)** T1-weighted MRI and PET results of case 1. No abnormality was found in MRI, while hypometabolism was identified in the right superior temporal gyrus. **(B)** Stereoelectroencephalography (SEEG) signals in the ictal period. Fast activities originated from the right superior temporal gyrus (N1-8) and spread rapidly to the right insular lobe (I3-8) and hippocampus (H1-3, B1-3). White arrow: the location of the electrodes N in MRI (right superior temporal gyrus).

**Figure 2:**
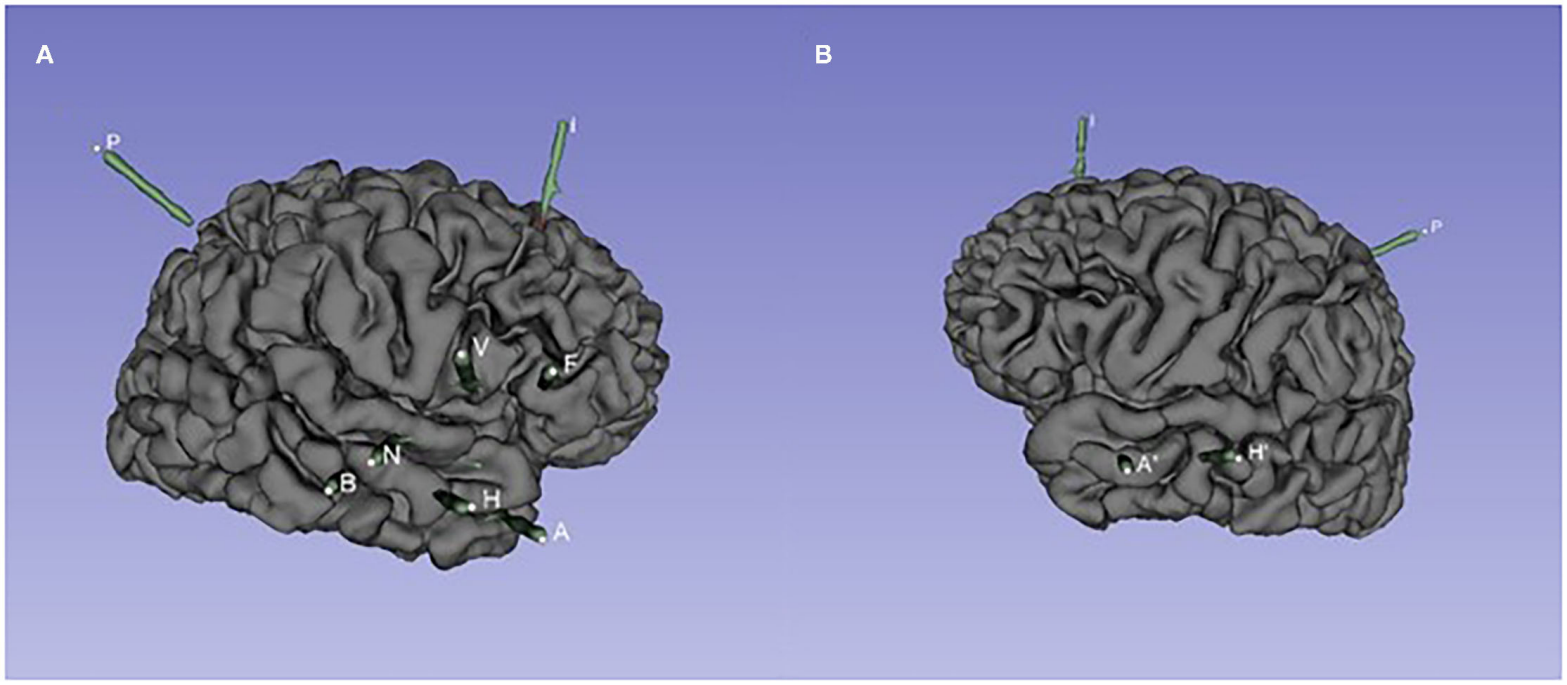
The spatial position of SEEG electrodes of case 1. **(A)** Right hemisphere. Entry points to target points: A: the middle temporal gyrus to the amygdala; H: the middle temporal gyrus to the head of the hippocampus; B: the middle temporal gyrus to the middle of the hippocampus; N: the superior temporal gyrus to the posterior insular gyrus; V: opercular partis to the short gyri of insula; F: pars triangularis to anterior cingulated cortex; I: the middle frontal gyrus to the accessory gyrus; P: the supramarginal gyrus to the anterior insular gyrus. **(B)** Left hemisphere. Entry points to target points: A′: the middle temporal gyrus to the amygdala; H′: the middle temporal gyrus to the head of the hippocampus.

No abnormality was found in 3.0T T1-weighted (voxel space = 2 × 2 × 2 mm^3^, TR = 381 ms, TE = 2.3 ms), T2-weighted (voxel space = 2 × 2 × 2 mm^3^, TR = 3,000 ms, TE = 100 ms) or fluid-attenuated inversion recovery (FLAIR) (voxel space=2 × 2 × 2 mm^3^, TR = 4,800 ms, TE = 411.7 ms) MRI. The ^18^F-fluorodeoxyglucose (^18^F-FDG) PET results were characterised by hypometabolism in the right superior temporal gyrus, right medial temporal lobe (Figure 1), and right insular lobe. The SEEG was performed, and the positions of electrodes were shown in Figure 2.

The SEEG in the interictal period showed obvious fast activities in the right superior temporal gyrus and the right anterior insular lobe. In the ictal period, fast activities originated from the right superior temporal gyrus (N1-8) and spread rapidly to the right anterior insular lobe (I3-8) and hippocampus (H1-3, B1-3) (Figure 1B).

**Figure 3:**
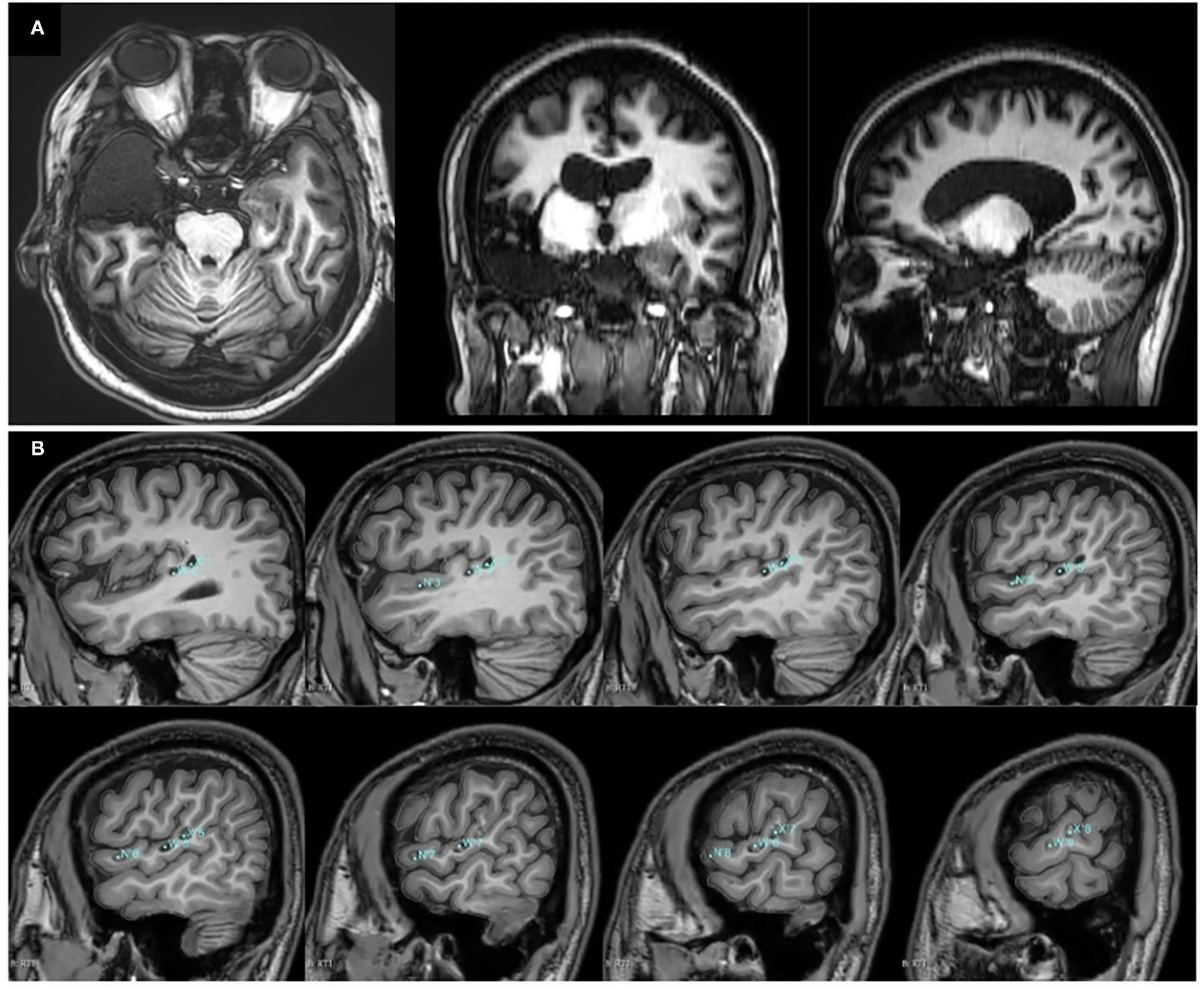
Postoperative T1-weighted MRI results. **(A)** T1-weighted MRI results of case 1 after the right anteromedial temporal lobe and insular lobe resection. **(B)** T1-weighted MRI results of case 2 after SEEG-guided radiofrequency thermocoagulation (RFTC). The locations and labels of the electrodes for the thermocoagulation were marked in blue.

The SEEG-guided RFTC was performed at electrodes N1-8 (Figure 1B), but there was no significant seizure decrease after the RFTC. She underwent the right anteromedial temporal lobe and insular lobe resection. The postoperative T1-weighted MRI results are shown in Figure 3A. Follow-up results showed that in the next 2 years, only one seizure occurred in 3 months after the operation.

### Case 2

Case 2 was a son of case 1, with normal delivery and no nervous system injury. The first seizure occurred at the age of 19, with limb tonic seizures at the frequency of about once a month. When he came to our hospital at 24, he felt dizzy and cannot understand other people's language, then, he experienced consciousness loss, bilateral upper limbs flexion, and elicited mouth movements. Genetic tests showed an abnormality in the microtubule-associated protein tau (MAPT) gene and linked it to chr17:44060593.

Scalp EEG showed that the epileptiform activities mainly occurred in the left sphenoidal electrode and frontotemporal region. Ictal EEG was characterised by initial attenuation, followed by rhythmic 5–6 Hz activities in the left sphenoidal electrode and frontotemporal region.

**Figure 4:**
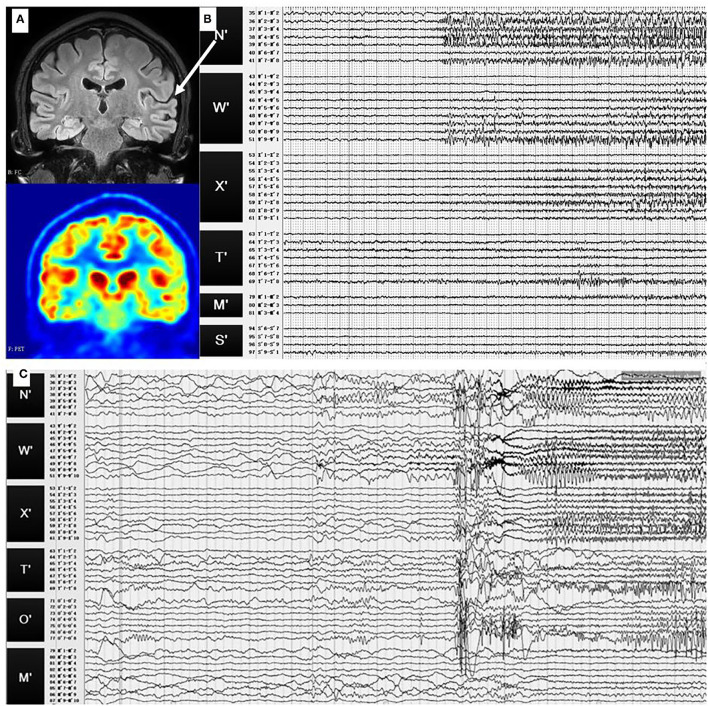
**(A)** Preoperative T1-weighted MRI and PET results of case 2. No abnormality was found in MRI, while hypometabolism was identified in the left superior temporal gyrus. **(B)** SEEG signals of the first seizure pattern. During this pattern, fast activities or sharp slow waves were identified at the left superior temporal gyrus (N′3-5), then sharp waves and spike waves spread in the left superior temporal gyrus (N′2-8, W′5-10, X′4-8). **(C)** SEEG signals of the second seizure pattern. There were fast activities and slow-wave oscillation at the left superior temporal gyrus (N′3-5), then the fast activities spread in the left superior temporal gyrus (W′2-9, N′2-6), and finally, spread to the other sites. White arrow: the location of the electrodes N′ in MRI (left superior temporal gyrus).

No abnormality was found in MRI results with the same scanning parameters as in case 1. The PET results suggested hypometabolism in the left superior temporal gyrus, left supramarginal gyrus, and inferior central gyrus (Figure 4A).

The SEEG in the interictal period showed obvious abnormal discharges in the left superior temporal gyrus, which then affected the lateral temporal lobe. Two types of seizure patterns were identified.

In the first seizure pattern, the patient felt dizzy and had auditory hallucinations. The seizures were then followed by daze, wink, and chewing. During this pattern, fast activities or sharp slow waves were identified at the left superior temporal gyrus (N'3-5), then, sharp waves and spike waves spread in the left superior temporal gyrus (N'2-8, W'5-10, X'4-8), as shown in Figure 4B.

The second seizure pattern was characterised by feeling uncomfortable, groaning, right and then both upper limbs tonic seizures, right-turning head, and, finally, secondarily generalised tonic-clonic seizures. There were fast activities and slow-wave oscillation at the left superior temporal gyrus (N'3-5), then, the fast activities spread in the left superior temporal gyrus (W'2-9, N'2-6) and, finally, spread to the other sites (Figure 4C).

The RFTC was performed at electrodes W'1-10, N'1-8, and X'1-10. The T1-weighted MRI results after the RFTC are shown in Figure 3B. According to the last follow-up at 18 months after the RFTC, he remained seizure-free (Engel 1A).

## Discussion

Familial temporal lobe epilepsy (FTLE) can be subdivided into lateral and mesial forms by clinical and genetic characteristics, and FLTLE is characterised by auditory auras (1). Several protein mutations were considered to be associated with temporal lobe epilepsy, such as axon guidance proteins, leucine-rich glioma inactivated 1 protein, microtubular protein, pore-forming, chromatin remodelling, and chemokine proteins (4). Hyperphosphorylated tau protein has been identified in patients with refractory temporal lobe epilepsy and might be the cause of the cognitive decline in these patients (5). Given that there were no other genetic abnormalities detected in case 2, we suppose that MAPT mutation played a role in the case of FLTLE. However, such supposition still needs further verification.

For patients with FTLE, most of the reports mentioned that they had a good response to anti-seizure medications, and the disease would be gradually controlled with the increase of age. Cendes et al. (6) reported 36 FTLE cases, including eight with refractory epilepsy who underwent surgery. Fabera et al. (7) also reported two FTLE cases that underwent left anteromedial temporal lobectomy. Koizumi et al. (8) reported five refractory epilepsy cases in one family. Among them, four patients underwent bilateral subdural electrode implantation and resection, two patients underwent standard anterior temporal lobectomy and two underwent lateral temporal lobectomy, and all of them were rated as Engel 1 after the operation. The cases reported in this paper also showed that surgery has a good effect on patients with refractory FLTLE.

According to the results of SEEG and clinical information, the superior temporal gyrus was confirmed as the onset area, and SEEG-guided RFTC was performed. However, only case 2 had a significant curative effect. The SEEG signal can provide effective information to locate the epileptogenic zone, but by the limited number of electrodes and the range of thermocoagulation, SEEG-guided RFTC may easily miss the epileptogenic zone. Koizumi et al. (8) reported two cases, whose epileptic focuses were located in the superior temporal gyrus by electrocorticographic results, one case in the lateral temporal lobe (including the superior temporal gyrus) and the other in the inferior temporal gyrus and fusiform gyrus. Whether these results suggest that the epileptic focus of FLTLE located in the superior temporal lobe still needs more clinical results to confirm. We identified three different seizure spreading patterns: (1) pure superior temporal gyrus with auditory symptoms; (2) mesial temporal lobe with automatisms; and (3) frontal lobe with movement symptoms.

Interestingly, the epileptic focus in case 1 is located in the right superior temporal gyrus, whereas the one in case 2 is located in the left superior temporal gyrus. In the FLTLE family reported by Koizumi et al., the three patients' epilepsy focuses were located on the left side and one on the right (8). The invasive EEG monitoring results suggest that, although FLTLE is an autosomal dominant genetic disease, the side of epileptic focus is not fixed.

## Data Availability Statement

The original contributions presented in the study are included in the article/supplementary material, further inquiries can be directed to the corresponding author/s.

## Ethics Statement

The studies involving human participants were reviewed and approved by the Ruijin Hospital Luwan Branch Ethics Committee, Shanghai Jiao Tong University School of Medicine. The patients/participants provided their written informed consent to participate in this study. Written informed consent was obtained from the individual(s) for the publication of any potentially identifiable images or data included in this article.

## Author Contributions

QL, XY, CW, and JX designed and implemented the treatment and provided clinical information. ZW, PZ, and JZ summarised relevant researches and wrote and revised the case report. All authors contributed to the article and approved the submitted version.

## Funding

This work was supported by National Natural Science Foundation of China (No. 82071550) and Shanghai Jiao Tong University Fund for Interdisciplinary Research for Medical Applications (YG2021QN30).

## Conflict of Interest

The authors declare that the research was conducted in the absence of any commercial or financial relationships that could be construed as a potential conflict of interest.

## Publisher's Note

All claims expressed in this article are solely those of the authors and do not necessarily represent those of their affiliated organizations, or those of the publisher, the editors and the reviewers. Any product that may be evaluated in this article, or claim that may be made by its manufacturer, is not guaranteed or endorsed by the publisher.
